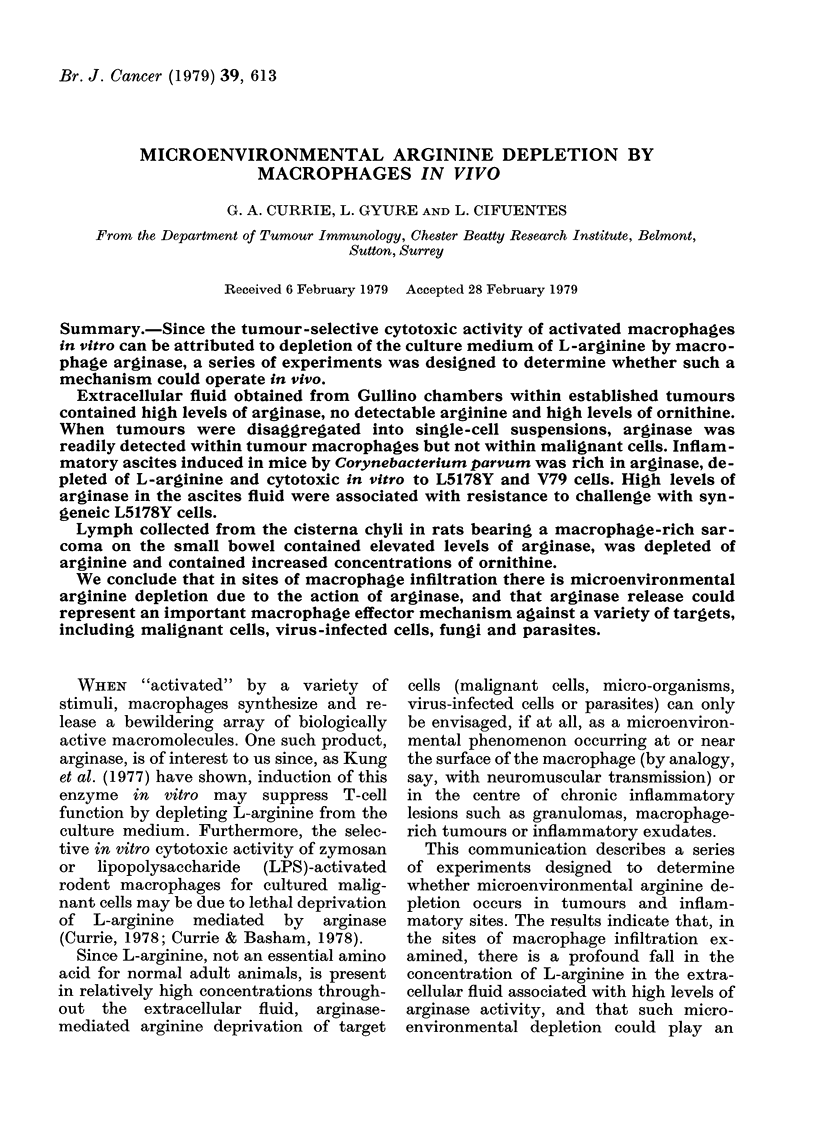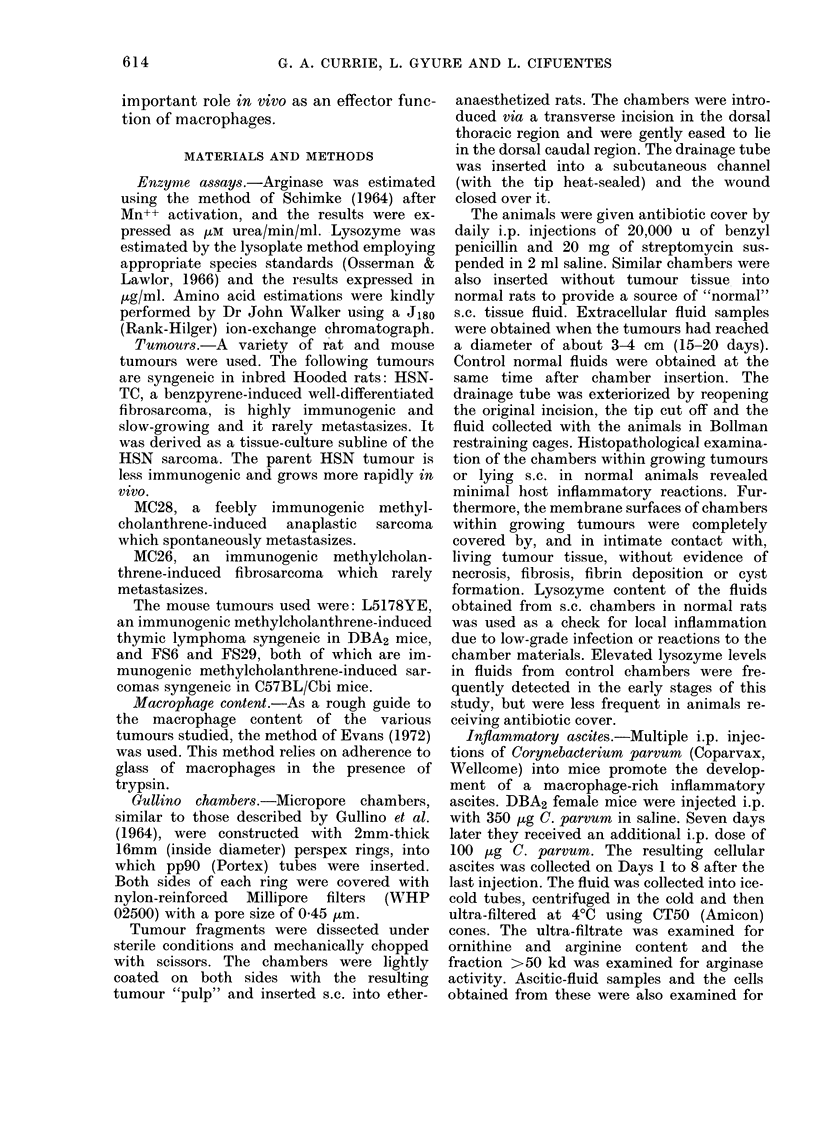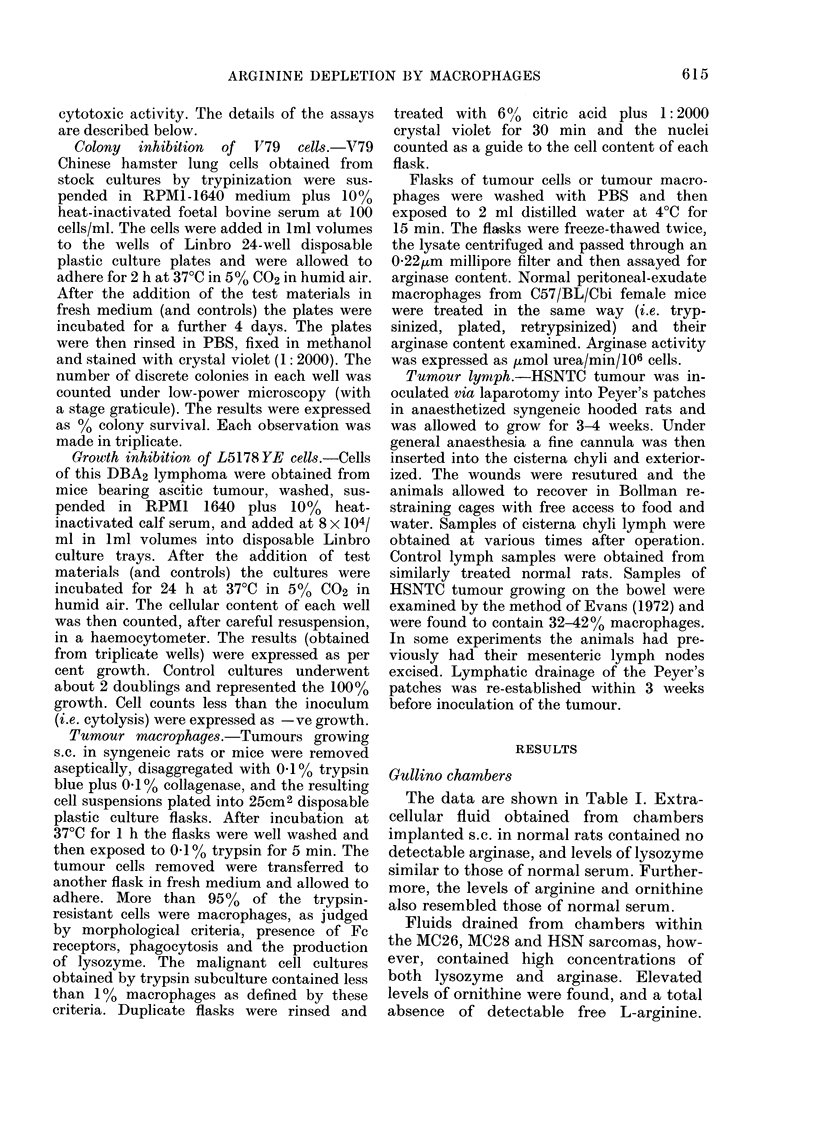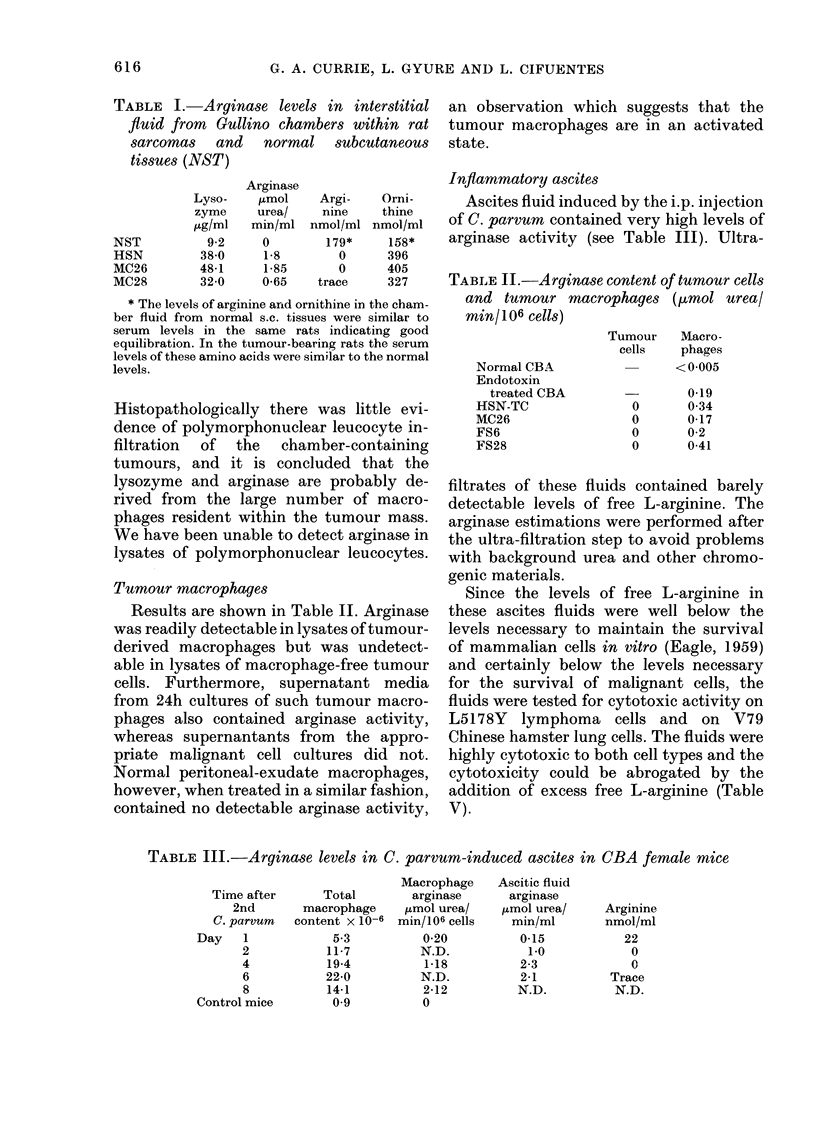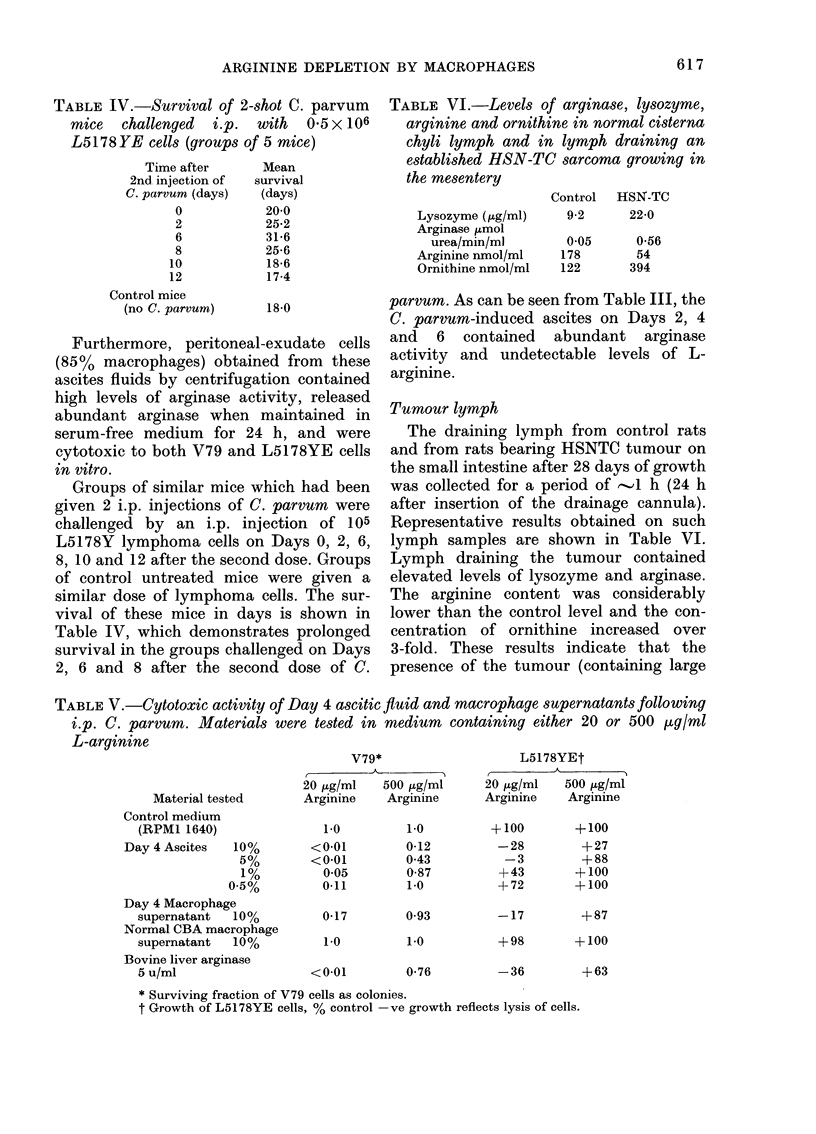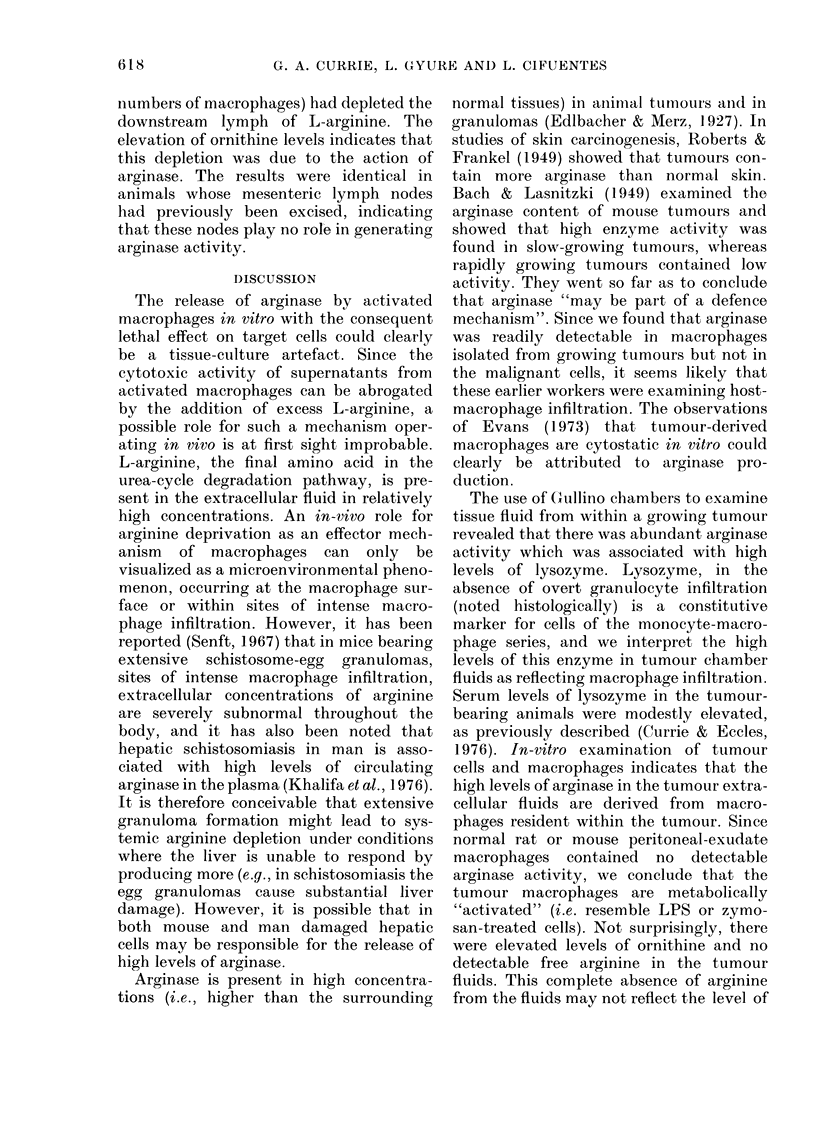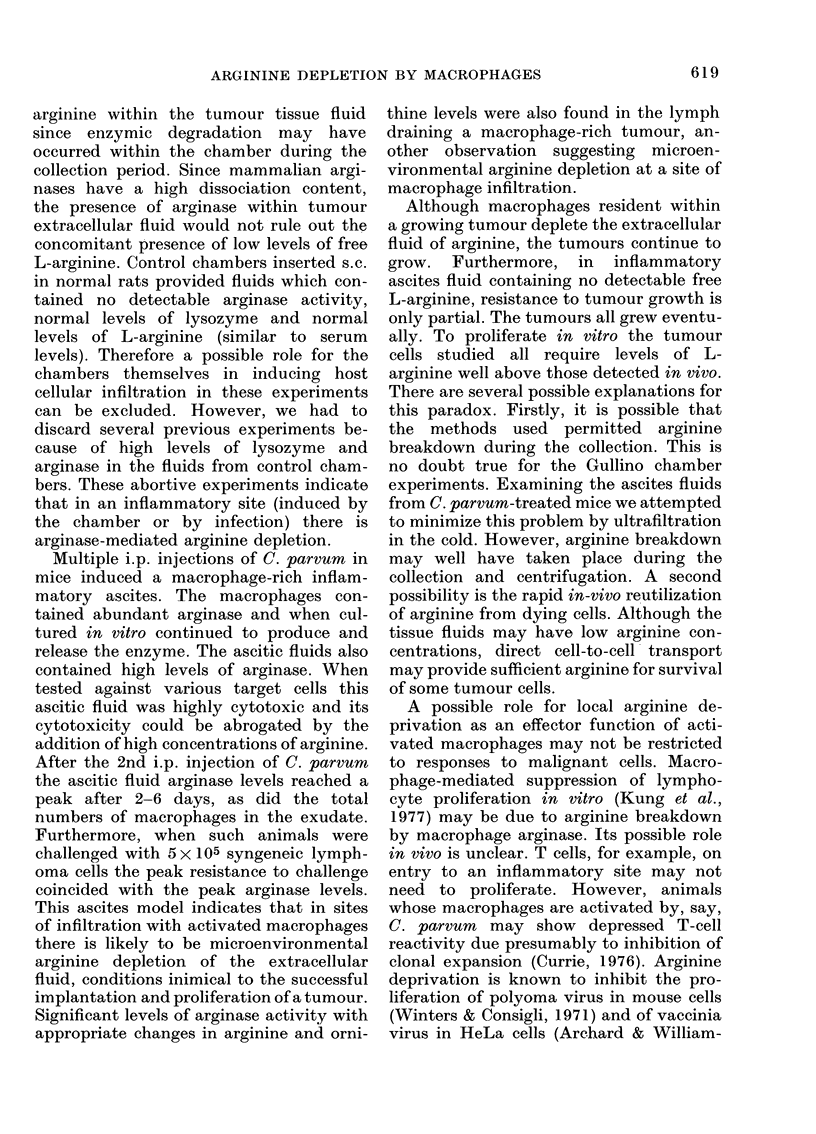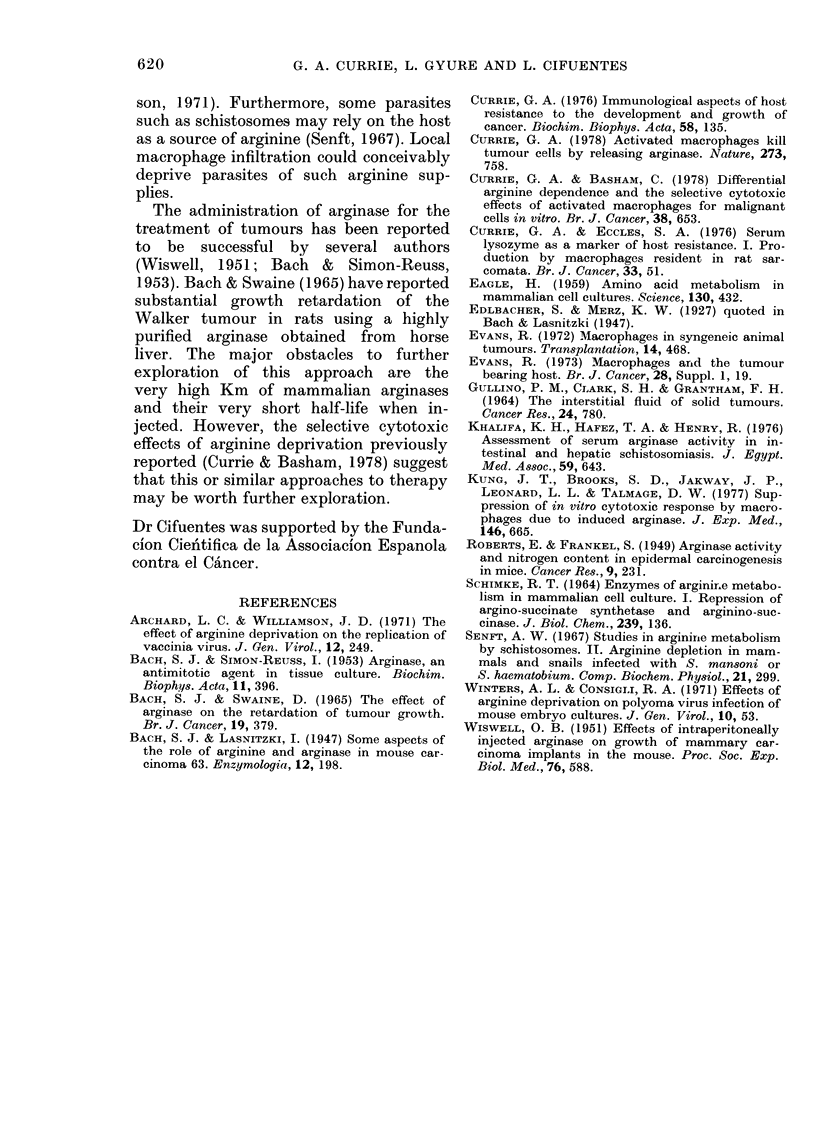# Microenvironmental arginine depletion by macrophages in vivo.

**DOI:** 10.1038/bjc.1979.112

**Published:** 1979-06

**Authors:** G. A. Currie, L. Gyure, L. Cifuentes

## Abstract

Since the tumour-selective cytotoxic activity of activated macrophages in vitro can be attributed to depletion of the culture medium of L-arginine by macrophage arginase, a series of experiments was designed to determine whether such a mechanism could operate in vivo. Extracellular fluid obtained from Gullino chambers within established tumours contained high levels of arginase, no detectable arginine and high levels of ornithine. When tumours were disaggregated into single-cell suspensions, arginase was readily detected within tumour macrophages but not within malignant cells. Inflammatory ascites induced in mice by Corynebacterium parvum was rich in arginase, depleted of L-arginine and cytotoxic in vitro to L5178Y and V79 cells. High levels of arginase in the ascites fluid were associated with resistance to challenge with syngeneic L5178Y cells. Lymph collected from the cisterna chyli in rats bearing a macrophage-rich sarcoma on the small bowel contained elevated levels of arginase, was depleted of arginine and contained increased concentrations of ornithine. We conclude that in sites of macrophage infiltration there is microenvironmental arginine depletion due to the action of arginase, and that arginase release could represent an important macrophage effector mechanism against a variety of targets, including malignant cells, virus-infected cells, fungi and parasites.


*Br. J. Cancer* (1979) **39**, 613

### MICROENVIRONMENTAL ARGININE DEPLETION BY MACROPHAGES *IN VIVO*

G. A. CURRIE, L. GYURE AND L. CIFUENTES

*From the Department of Tumour Immunology, Chester Beatty Research Institute, Belmont, Sutton, Surrey*

Received 6 February 1979 Accepted 28 February 1979

**Summary.—Since the tumour-selective cytotoxic activity of activated macrophages *in vitro* can be attributed to depletion of the culture medium of L-arginine by macrophage arginase, a series of experiments was designed to determine whether such a mechanism could operate *in vivo*.**

**Extracellular fluid obtained from Gullino chambers within established tumours contained high levels of arginase, no detectable arginine and high levels of ornithine. When tumours were disaggregated into single-cell suspensions, arginase was readily detected within tumour macrophages but not within malignant cells. Inflammatory ascites induced in mice by *Corynebacterium parvum* was rich in arginase, depleted of L-arginine and cytotoxic *in vitro* to L5178Y and V79 cells. High levels of arginase in the ascites fluid were associated with resistance to challenge with syngeneic L5178Y cells.**

**Lymph collected from the cisterna chyli in rats bearing a macrophage-rich sarcoma on the small bowel contained elevated levels of arginase, was depleted of arginine and contained increased concentrations of ornithine.**

**We conclude that in sites of macrophage infiltration there is microenvironmental arginine depletion due to the action of arginase, and that arginase release could represent an important macrophage effector mechanism against a variety of targets, including malignant cells, virus-infected cells, fungi and parasites.**

WHEN "activated" by a variety of stimuli, macrophages synthesize and release a bewildering array of biologically active macromolecules. One such product, arginase, is of interest to us since, as Kung *et al.* (1977) have shown, induction of this enzyme *in vitro* may suppress T-cell function by depleting L-arginine from the culture medium. Furthermore, the selective *in vitro* cytotoxic activity of zymosan or lipopolysaccharide (LPS)-activated rodent macrophages for cultured malignant cells may be due to lethal deprivation of L-arginine mediated by arginase (Currie, 1978; Currie & Basham, 1978).

Since L-arginine, not an essential amino acid for normal adult animals, is present in relatively high concentrations throughout the extracellular fluid, arginase-mediated arginine deprivation of target cells (malignant cells, micro-organisms, virus-infected cells or parasites) can only be envisaged, if at all, as a microenvironmental phenomenon occurring at or near the surface of the macrophage (by analogy, say, with neuromuscular transmission) or in the centre of chronic inflammatory lesions such as granulomas, macrophage-rich tumours or inflammatory exudates.

This communication describes a series of experiments designed to determine whether microenvironmental arginine depletion occurs in tumours and inflammatory sites. The results indicate that, in the sites of macrophage infiltration examined, there is a profound fall in the concentration of L-arginine in the extracellular fluid associated with high levels of arginase activity, and that such microenvironmental depletion could play an important role *in vivo* as an effector function of macrophages.

### MATERIALS AND METHODS

*Enzyme assays.*—Arginase was estimated using the method of Schimke (1964) after $Mn^{++}$ activation, and the results were expressed as $\mu$M urea/min/ml. Lysozyme was estimated by the lysoplate method employing appropriate species standards (Osserman & Lawlor, 1966) and the results expressed in $\mu$g/ml. Amino acid estimations were kindly performed by Dr John Walker using a $J_{180}$ (Rank-Hilger) ion-exchange chromatograph.

*Tumours.*—A variety of rat and mouse tumours were used. The following tumours are syngeneic in inbred Hooded rats: HSN-TC, a benzpyrene-induced well-differentiated fibrosarcoma, is highly immunogenic and slow-growing and it rarely metastasizes. It was derived as a tissue-culture subline of the HSN sarcoma. The parent HSN tumour is less immunogenic and grows more rapidly *in vivo*.

MC28, a feebly immunogenic methylcholanthrene-induced anaplastic sarcoma which spontaneously metastasizes.

MC26, an immunogenic methylcholanthrene-induced fibrosarcoma which rarely metastasizes.

The mouse tumours used were: L5178YE, an immunogenic methylcholanthrene-induced thymic lymphoma syngeneic in $DBA_2$ mice, and FS6 and FS29, both of which are immunogenic methylcholanthrene-induced sarcomas syngeneic in C57BL/Cbi mice.

*Macrophage content.*—As a rough guide to the macrophage content of the various tumours studied, the method of Evans (1972) was used. This method relies on adherence to glass of macrophages in the presence of trypsin.

*Gullino chambers.*—Micropore chambers, similar to those described by Gullino *et al.* (1964), were constructed with 2mm-thick 16mm (inside diameter) perspex rings, into which pp90 (Portex) tubes were inserted. Both sides of each ring were covered with nylon-reinforced Millipore filters (WHP 02500) with a pore size of 0·45 $\mu$m.

Tumour fragments were dissected under sterile conditions and mechanically chopped with scissors. The chambers were lightly coated on both sides with the resulting tumour "pulp" and inserted s.c. into ether-anaesthetized rats. The chambers were introduced *via* a transverse incision in the dorsal thoracic region and were gently eased to lie in the dorsal caudal region. The drainage tube was inserted into a subcutaneous channel (with the tip heat-sealed) and the wound closed over it.

The animals were given antibiotic cover by daily i.p. injections of 20,000 u of benzyl penicillin and 20 mg of streptomycin suspended in 2 ml saline. Similar chambers were also inserted without tumour tissue into normal rats to provide a source of "normal" s.c. tissue fluid. Extracellular fluid samples were obtained when the tumours had reached a diameter of about 3–4 cm (15–20 days). Control normal fluids were obtained at the same time after chamber insertion. The drainage tube was exteriorized by reopening the original incision, the tip cut off and the fluid collected with the animals in Bollman restraining cages. Histopathological examination of the chambers within growing tumours or lying s.c. in normal animals revealed minimal host inflammatory reactions. Furthermore, the membrane surfaces of chambers within growing tumours were completely covered by, and in intimate contact with, living tumour tissue, without evidence of necrosis, fibrosis, fibrin deposition or cyst formation. Lysozyme content of the fluids obtained from s.c. chambers in normal rats was used as a check for local inflammation due to low-grade infection or reactions to the chamber materials. Elevated lysozyme levels in fluids from control chambers were frequently detected in the early stages of this study, but were less frequent in animals receiving antibiotic cover.

*Inflammatory ascites.*—Multiple i.p. injections of *Corynebacterium parvum* (Coparvax, Wellcome) into mice promote the development of a macrophage-rich inflammatory ascites. $DBA_2$ female mice were injected i.p. with 350 $\mu$g *C. parvum* in saline. Seven days later they received an additional i.p. dose of 100 $\mu$g *C. parvum.* The resulting cellular ascites was collected on Days 1 to 8 after the last injection. The fluid was collected into ice-cold tubes, centrifuged in the cold and then ultra-filtered at 4°C using CT50 (Amicon) cones. The ultra-filtrate was examined for ornithine and arginine content and the fraction $>50$ kd was examined for arginase activity. Ascitic-fluid samples and the cells obtained from these were also examined for cytotoxic activity. The details of the assays are described below.

*Colony inhibition of V79 cells.*—V79 Chinese hamster lung cells obtained from stock cultures by trypinization were suspended in RPMI-1640 medium plus 10% heat-inactivated foetal bovine serum at 100 cells/ml. The cells were added in 1ml volumes to the wells of Linbro 24-well disposable plastic culture plates and were allowed to adhere for 2 h at 37°C in 5% $CO_2$ in humid air. After the addition of the test materials in fresh medium (and controls) the plates were incubated for a further 4 days. The plates were then rinsed in PBS, fixed in methanol and stained with crystal violet (1:2000). The number of discrete colonies in each well was counted under low-power microscopy (with a stage graticule). The results were expressed as % colony survival. Each observation was made in triplicate.

*Growth inhibition of L5178YE cells.*—Cells of this $DBA_2$ lymphoma were obtained from mice bearing ascitic tumour, washed, suspended in RPMI 1640 plus 10% heat-inactivated calf serum, and added at $8 \times 10^4$/ml in 1ml volumes into disposable Linbro culture trays. After the addition of test materials (and controls) the cultures were incubated for 24 h at 37°C in 5% $CO_2$ in humid air. The cellular content of each well was then counted, after careful resuspension, in a haemocytometer. The results (obtained from triplicate wells) were expressed as per cent growth. Control cultures underwent about 2 doublings and represented the 100% growth. Cell counts less than the inoculum (*i.e.* cytolysis) were expressed as −ve growth.

*Tumour macrophages.*—Tumours growing s.c. in syngeneic rats or mice were removed aseptically, disaggregated with 0·1% trypsin blue plus 0·1% collagenase, and the resulting cell suspensions plated into 25cm$^2$ disposable plastic culture flasks. After incubation at 37°C for 1 h the flasks were well washed and then exposed to 0·1% trypsin for 5 min. The tumour cells removed were transferred to another flask in fresh medium and allowed to adhere. More than 95% of the trypsin-resistant cells were macrophages, as judged by morphological criteria, presence of Fc receptors, phagocytosis and the production of lysozyme. The malignant cell cultures obtained by trypsin subculture contained less than 1% macrophages as defined by these criteria. Duplicate flasks were rinsed and treated with 6% citric acid plus 1:2000 crystal violet for 30 min and the nuclei counted as a guide to the cell content of each flask.

Flasks of tumour cells or tumour macrophages were washed with PBS and then exposed to 2 ml distilled water at 4°C for 15 min. The flasks were freeze-thawed twice, the lysate centrifuged and passed through an 0·22$\mu$m millipore filter and then assayed for arginase content. Normal peritoneal-exudate macrophages from C57/BL/Cbi female mice were treated in the same way (*i.e.* trypsinized, plated, retrypsinized) and their arginase content examined. Arginase activity was expressed as $\mu$mol urea/min/$10^6$ cells.

*Tumour lymph.*—HSNTC tumour was inoculated *via* laparotomy into Peyer's patches in anaesthetized syngeneic hooded rats and was allowed to grow for 3–4 weeks. Under general anaesthesia a fine cannula was then inserted into the cisterna chyli and exteriorized. The wounds were resutured and the animals allowed to recover in Bollman restraining cages with free access to food and water. Samples of cisterna chyli lymph were obtained at various times after operation. Control lymph samples were obtained from similarly treated normal rats. Samples of HSNTC tumour growing on the bowel were examined by the method of Evans (1972) and were found to contain 32–42% macrophages. In some experiments the animals had previously had their mesenteric lymph nodes excised. Lymphatic drainage of the Peyer's patches was re-established within 3 weeks before inoculation of the tumour.

### RESULTS

### *Gullino chambers*

The data are shown in Table I. Extracellular fluid obtained from chambers implanted s.c. in normal rats contained no detectable arginase, and levels of lysozyme similar to those of normal serum. Furthermore, the levels of arginine and ornithine also resembled those of normal serum.

Fluids drained from chambers within the MC26, MC28 and HSN sarcomas, however, contained high concentrations of both lysozyme and arginase. Elevated levels of ornithine were found, and a total absence of detectable free L-arginine.

TABLE I.—*Arginase levels in interstitial fluid from Gullino chambers within rat sarcomas and normal subcutaneous tissues (NST)*

| | Lysozyme μg/ml | Arginase μmol urea/min/ml | Arginine nmol/ml | Ornithine nmol/ml |
|---|---|---|---|---|
| NST | 9·2 | 0 | 179* | 158* |
| HSN | 38·0 | 1·8 | 0 | 396 |
| MC26 | 48·1 | 1·85 | 0 | 405 |
| MC28 | 32·0 | 0·65 | trace | 327 |

* The levels of arginine and ornithine in the chamber fluid from normal s.c. tissues were similar to serum levels in the same rats indicating good equilibration. In the tumour-bearing rats the serum levels of these amino acids were similar to the normal levels.

Histopathologically there was little evidence of polymorphonuclear leucocyte infiltration of the chamber-containing tumours, and it is concluded that the lysozyme and arginase are probably derived from the large number of macrophages resident within the tumour mass. We have been unable to detect arginase in lysates of polymorphonuclear leucocytes.

*Tumour macrophages*

Results are shown in Table II. Arginase was readily detectable in lysates of tumour-derived macrophages but was undetectable in lysates of macrophage-free tumour cells. Furthermore, supernatant media from 24h cultures of such tumour macrophages also contained arginase activity, whereas supernantants from the appropriate malignant cell cultures did not. Normal peritoneal-exudate macrophages, however, when treated in a similar fashion, contained no detectable arginase activity, an observation which suggests that the tumour macrophages are in an activated state.

*Inflammatory ascites*

Ascites fluid induced by the i.p. injection of *C. parvum* contained very high levels of arginase activity (see Table III). Ultra-filtrates of these fluids contained barely detectable levels of free L-arginine. The arginase estimations were performed after the ultra-filtration step to avoid problems with background urea and other chromogenic materials.

TABLE II.—*Arginase content of tumour cells and tumour macrophages ($\mu$mol urea/min/10$^6$ cells)*

| | Tumour cells | Macrophages |
|---|---|---|
| Normal CBA | — | <0·005 |
| Endotoxin treated CBA | — | 0·19 |
| HSN-TC | 0 | 0·34 |
| MC26 | 0 | 0·17 |
| FS6 | 0 | 0·2 |
| FS28 | 0 | 0·41 |

Since the levels of free L-arginine in these ascites fluids were well below the levels necessary to maintain the survival of mammalian cells *in vitro* (Eagle, 1959) and certainly below the levels necessary for the survival of malignant cells, the fluids were tested for cytotoxic activity on L5178Y lymphoma cells and on V79 Chinese hamster lung cells. The fluids were highly cytotoxic to both cell types and the cytotoxicity could be abrogated by the addition of excess free L-arginine (Table V).

TABLE III.—*Arginase levels in C. parvum-induced ascites in CBA female mice*

| Time after 2nd *C. parvum* | Total macrophage content $\times 10^{-6}$ | Macrophage arginase μmol urea/min/10$^6$ cells | Ascitic fluid arginase μmol urea/min/ml | Arginine nmol/ml |
|---|---|---|---|---|
| Day 1 | 5·3 | 0·20 | 0·15 | 22 |
| 2 | 11·7 | N.D. | 1·0 | 0 |
| 4 | 19·4 | 1·18 | 2·3 | 0 |
| 6 | 22·0 | N.D. | 2·1 | Trace |
| 8 | 14·1 | 2·12 | N.D. | N.D. |
| Control mice | 0·9 | 0 | | |

TABLE IV.—*Survival of 2-shot* C. parvum *mice challenged i.p. with* $0 \cdot 5 \times 10^6$ *L5178YE cells (groups of 5 mice)*

| Time after 2nd injection of *C. parvum* (days) | Mean survival (days) |
|---|---|
| 0 | 20·0 |
| 2 | 25·2 |
| 6 | 31·6 |
| 8 | 25·6 |
| 10 | 18·6 |
| 12 | 17·4 |
| Control mice (no *C. parvum*) | 18·0 |

Furthermore, peritoneal-exudate cells (85% macrophages) obtained from these ascites fluids by centrifugation contained high levels of arginase activity, released abundant arginase when maintained in serum-free medium for 24 h, and were cytotoxic to both V79 and L5178YE cells *in vitro*.

Groups of similar mice which had been given 2 i.p. injections of *C. parvum* were challenged by an i.p. injection of $10^5$ L5178Y lymphoma cells on Days 0, 2, 6, 8, 10 and 12 after the second dose. Groups of control untreated mice were given a similar dose of lymphoma cells. The survival of these mice in days is shown in Table IV, which demonstrates prolonged survival in the groups challenged on Days 2, 6 and 8 after the second dose of *C. parvum*. As can be seen from Table III, the *C. parvum*-induced ascites on Days 2, 4 and 6 contained abundant arginase activity and undetectable levels of L-arginine.

TABLE VI.—*Levels of arginase, lysozyme, arginine and ornithine in normal cisterna chyli lymph and in lymph draining an established HSN-TC sarcoma growing in the mesentery*

| | Control | HSN-TC |
|---|---|---|
| Lysozyme (μg/ml) | 9·2 | 22·0 |
| Arginase μmol urea/min/ml | 0·05 | 0·56 |
| Arginine nmol/ml | 178 | 54 |
| Ornithine nmol/ml | 122 | 394 |

*Tumour lymph*

The draining lymph from control rats and from rats bearing HSNTC tumour on the small intestine after 28 days of growth was collected for a period of ~1 h (24 h after insertion of the drainage cannula). Representative results obtained on such lymph samples are shown in Table VI. Lymph draining the tumour contained elevated levels of lysozyme and arginase. The arginine content was considerably lower than the control level and the concentration of ornithine increased over 3-fold. These results indicate that the presence of the tumour (containing large

TABLE V.—*Cytotoxic activity of Day* 4 *ascitic fluid and macrophage supernatants following i.p. C. parvum. Materials were tested in medium containing either* 20 *or* 500 *μg/ml L-arginine*

| Material tested | V79* 20 μg/ml Arginine | V79* 500 μg/ml Arginine | L5178YE† 20 μg/ml Arginine | L5178YE† 500 μg/ml Arginine |
|---|---|---|---|---|
| Control medium (RPMI 1640) | 1·0 | 1·0 | +100 | +100 |
| Day 4 Ascites 10% | <0·01 | 0·12 | −28 | +27 |
| 5% | <0·01 | 0·43 | −3 | +88 |
| 1% | 0·05 | 0·87 | +43 | +100 |
| 0·5% | 0·11 | 1·0 | +72 | +100 |
| Day 4 Macrophage supernatant 10% | 0·17 | 0·93 | −17 | +87 |
| Normal CBA macrophage supernatant 10% | 1·0 | 1·0 | +98 | +100 |
| Bovine liver arginase 5 u/ml | <0·01 | 0·76 | −36 | +63 |

* Surviving fraction of V79 cells as colonies.
† Growth of L5178YE cells, % control —ve growth reflects lysis of cells.

numbers of macrophages) had depleted the downstream lymph of L-arginine. The elevation of ornithine levels indicates that this depletion was due to the action of arginase. The results were identical in animals whose mesenteric lymph nodes had previously been excised, indicating that these nodes play no role in generating arginase activity.

### DISCUSSION

The release of arginase by activated macrophages *in vitro* with the consequent lethal effect on target cells could clearly be a tissue-culture artefact. Since the cytotoxic activity of supernatants from activated macrophages can be abrogated by the addition of excess L-arginine, a possible role for such a mechanism operating *in vivo* is at first sight improbable. L-arginine, the final amino acid in the urea-cycle degradation pathway, is present in the extracellular fluid in relatively high concentrations. An *in-vivo* role for arginine deprivation as an effector mechanism of macrophages can only be visualized as a microenvironmental phenomenon, occurring at the macrophage surface or within sites of intense macrophage infiltration. However, it has been reported (Senft, 1967) that in mice bearing extensive schistosome-egg granulomas, sites of intense macrophage infiltration, extracellular concentrations of arginine are severely subnormal throughout the body, and it has also been noted that hepatic schistosomiasis in man is associated with high levels of circulating arginase in the plasma (Khalifa *et al.*, 1976). It is therefore conceivable that extensive granuloma formation might lead to systemic arginine depletion under conditions where the liver is unable to respond by producing more (*e.g.*, in schistosomiasis the egg granulomas cause substantial liver damage). However, it is possible that in both mouse and man damaged hepatic cells may be responsible for the release of high levels of arginase.

Arginase is present in high concentrations (*i.e.*, higher than the surrounding normal tissues) in animal tumours and in granulomas (Edlbacher & Merz, 1927). In studies of skin carcinogenesis, Roberts & Frankel (1949) showed that tumours contain more arginase than normal skin. Bach & Lasnitzki (1949) examined the arginase content of mouse tumours and showed that high enzyme activity was found in slow-growing tumours, whereas rapidly growing tumours contained low activity. They went so far as to conclude that arginase "may be part of a defence mechanism". Since we found that arginase was readily detectable in macrophages isolated from growing tumours but not in the malignant cells, it seems likely that these earlier workers were examining host-macrophage infiltration. The observations of Evans (1973) that tumour-derived macrophages are cytostatic *in vitro* could clearly be attributed to arginase production.

The use of Gullino chambers to examine tissue fluid from within a growing tumour revealed that there was abundant arginase activity which was associated with high levels of lysozyme. Lysozyme, in the absence of overt granulocyte infiltration (noted histologically) is a constitutive marker for cells of the monocyte-macrophage series, and we interpret the high levels of this enzyme in tumour chamber fluids as reflecting macrophage infiltration. Serum levels of lysozyme in the tumour-bearing animals were modestly elevated, as previously described (Currie & Eccles, 1976). *In-vitro* examination of tumour cells and macrophages indicates that the high levels of arginase in the tumour extracellular fluids are derived from macrophages resident within the tumour. Since normal rat or mouse peritoneal-exudate macrophages contained no detectable arginase activity, we conclude that the tumour macrophages are metabolically "activated" (*i.e.* resemble LPS or zymosan-treated cells). Not surprisingly, there were elevated levels of ornithine and no detectable free arginine in the tumour fluids. This complete absence of arginine from the fluids may not reflect the level of arginine within the tumour tissue fluid since enzymic degradation may have occurred within the chamber during the collection period. Since mammalian arginases have a high dissociation content, the presence of arginase within tumour extracellular fluid would not rule out the concomitant presence of low levels of free L-arginine. Control chambers inserted s.c. in normal rats provided fluids which contained no detectable arginase activity, normal levels of lysozyme and normal levels of L-arginine (similar to serum levels). Therefore a possible role for the chambers themselves in inducing host cellular infiltration in these experiments can be excluded. However, we had to discard several previous experiments because of high levels of lysozyme and arginase in the fluids from control chambers. These abortive experiments indicate that in an inflammatory site (induced by the chamber or by infection) there is arginase-mediated arginine depletion.

Multiple i.p. injections of *C. parvum* in mice induced a macrophage-rich inflammatory ascites. The macrophages contained abundant arginase and when cultured *in vitro* continued to produce and release the enzyme. The ascitic fluids also contained high levels of arginase. When tested against various target cells this ascitic fluid was highly cytotoxic and its cytotoxicity could be abrogated by the addition of high concentrations of arginine. After the 2nd i.p. injection of *C. parvum* the ascitic fluid arginase levels reached a peak after 2–6 days, as did the total numbers of macrophages in the exudate. Furthermore, when such animals were challenged with $5 \times 10^5$ syngeneic lymphoma cells the peak resistance to challenge coincided with the peak arginase levels. This ascites model indicates that in sites of infiltration with activated macrophages there is likely to be microenvironmental arginine depletion of the extracellular fluid, conditions inimical to the successful implantation and proliferation of a tumour. Significant levels of arginase activity with appropriate changes in arginine and ornithine levels were also found in the lymph draining a macrophage-rich tumour, another observation suggesting microenvironmental arginine depletion at a site of macrophage infiltration.

Although macrophages resident within a growing tumour deplete the extracellular fluid of arginine, the tumours continue to grow. Furthermore, in inflammatory ascites fluid containing no detectable free L-arginine, resistance to tumour growth is only partial. The tumours all grew eventually. To proliferate *in vitro* the tumour cells studied all require levels of L-arginine well above those detected *in vivo*. There are several possible explanations for this paradox. Firstly, it is possible that the methods used permitted arginine breakdown during the collection. This is no doubt true for the Gullino chamber experiments. Examining the ascites fluids from *C. parvum*-treated mice we attempted to minimize this problem by ultrafiltration in the cold. However, arginine breakdown may well have taken place during the collection and centrifugation. A second possibility is the rapid *in-vivo* reutilization of arginine from dying cells. Although the tissue fluids may have low arginine concentrations, direct cell-to-cell transport may provide sufficient arginine for survival of some tumour cells.

A possible role for local arginine deprivation as an effector function of activated macrophages may not be restricted to responses to malignant cells. Macrophage-mediated suppression of lymphocyte proliferation *in vitro* (Kung *et al.*, 1977) may be due to arginine breakdown by macrophage arginase. Its possible role *in vivo* is unclear. T cells, for example, on entry to an inflammatory site may not need to proliferate. However, animals whose macrophages are activated by, say, *C. parvum* may show depressed T-cell reactivity due presumably to inhibition of clonal expansion (Currie, 1976). Arginine deprivation is known to inhibit the proliferation of polyoma virus in mouse cells (Winters & Consigli, 1971) and of vaccinia virus in HeLa cells (Archard & Williamson, 1971). Furthermore, some parasites such as schistosomes may rely on the host as a source of arginine (Senft, 1967). Local macrophage infiltration could conceivably deprive parasites of such arginine supplies.

The administration of arginase for the treatment of tumours has been reported to be successful by several authors (Wiswell, 1951; Bach & Simon-Reuss, 1953). Bach & Swaine (1965) have reported substantial growth retardation of the Walker tumour in rats using a highly purified arginase obtained from horse liver. The major obstacles to further exploration of this approach are the very high Km of mammalian arginases and their very short half-life when injected. However, the selective cytotoxic effects of arginine deprivation previously reported (Currie & Basham, 1978) suggest that this or similar approaches to therapy may be worth further exploration.

Dr Cifuentes was supported by the Fundacíon Cieńtifica de la Associacíon Espanola contra el Cáncer.

### REFERENCES

ARCHARD, L. C. & WILLIAMSON, J. D. (1971) The effect of arginine deprivation on the replication of vaccinia virus. *J. Gen. Virol.*, **12**, 249.

BACH, S. J. & SIMON-REUSS, I. (1953) Arginase, an antimitotic agent in tissue culture. *Biochim. Biophys. Acta*, **11**, 396.

BACH, S. J. & SWAINE, D. (1965) The effect of arginase on the retardation of tumour growth. *Br. J. Cancer*, **19**, 379.

BACH, S. J. & LASNITZKI, I. (1947) Some aspects of the role of arginine and arginase in mouse carcinoma 63. *Enzymologia*, **12**, 198.

CURRIE, G. A. (1976) Immunological aspects of host resistance to the development and growth of cancer. *Biochim. Biophys. Acta*, **58**, 135.

CURRIE, G. A. (1978) Activated macrophages kill tumour cells by releasing arginase. *Nature*, **273**, 758.

CURRIE, G. A. & BASHAM, C. (1978) Differential arginine dependence and the selective cytotoxic effects of activated macrophages for malignant cells *in vitro*. *Br. J. Cancer*, **38**, 653.

CURRIE, G. A. & ECCLES, S. A. (1976) Serum lysozyme as a marker of host resistance. I. Production by macrophages resident in rat sarcomata. *Br. J. Cancer*, **33**, 51.

EAGLE, H. (1959) Amino acid metabolism in mammalian cell cultures. *Science*, **130**, 432.

EDLBACHER, S. & MERZ, K. W. (1927) quoted in Bach & Lasnitzki (1947).

EVANS, R. (1972) Macrophages in syngeneic animal tumours. *Transplantation*, **14**, 468.

EVANS, R. (1973) Macrophages and the tumour bearing host. *Br. J. Cancer*, **28**, Suppl. 1, 19.

GULLINO, P. M., CLARK, S. H. & GRANTHAM, F. H. (1964) The interstitial fluid of solid tumours. *Cancer Res.*, **24**, 780.

KHALIFA, K. H., HAFEZ, T. A. & HENRY, R. (1976) Assessment of serum arginase activity in intestinal and hepatic schistosomiasis. *J. Egypt. Med. Assoc.*, **59**, 643.

KUNG, J. T., BROOKS, S. D., JAKWAY, J. P., LEONARD, L. L. & TALMAGE, D. W. (1977) Suppression of *in vitro* cytotoxic response by macrophages due to induced arginase. *J. Exp. Med.*, **146**, 665.

ROBERTS, E. & FRANKEL, S. (1949) Arginase activity and nitrogen content in epidermal carcinogenesis in mice. *Cancer Res.*, **9**, 231.

SCHIMKE, R. T. (1964) Enzymes of arginine metabolism in mammalian cell culture. I. Repression of argino-succinate synthetase and arginino-succinase. *J. Biol. Chem.*, **239**, 136.

SENFT, A. W. (1967) Studies in arginine metabolism by schistosomes. II. Arginine depletion in mammals and snails infected with *S. mansoni* or *S. haematobium*. *Comp. Biochem. Physiol.*, **21**, 299.

WINTERS, A. L. & CONSIGLI, R. A. (1971) Effects of arginine deprivation on polyoma virus infection of mouse embryo cultures. *J. Gen. Virol.*, **10**, 53.

WISWELL, O. B. (1951) Effects of intraperitoneally injected arginase on growth of mammary carcinoma implants in the mouse. *Proc. Soc. Exp. Biol. Med.*, **76**, 588.